# Predicting rifampicin resistance in *Mycobacterium tuberculosis* using machine learning informed by protein structural and chemical features

**DOI:** 10.1183/23120541.00952-2024

**Published:** 2025-06-30

**Authors:** Charlotte I. Lynch, Dylan Adlard, Philip W. Fowler

**Affiliations:** 1 Nuffield Department of Medicine, University of Oxford, Oxford, UK; 2 National Institute of Health Research Oxford Biomedical Research Centre, John Radcliffe Hospital, Oxford, UK; 3 Health Protection Research Unit in Healthcare Associated Infections and Antimicrobial Resistance, University of Oxford, Oxford, UK; 4 These authors contributed equally

## Abstract

**Background:**

Rifampicin remains a key antibiotic in the treatment of tuberculosis. Despite advances in cataloguing resistance-associated variants (RAVs), novel and rare mutations in the relevant gene, *rpoB*, will be encountered in clinical samples, complicating the task of using genetics to predict whether a sample is resistant or not to rifampicin. We have trained a series of machine learning models with the aim of complementing genetics-based drug susceptibility testing.

**Methods:**

We built a Test+Train dataset comprising 219 susceptible mutations and 46 RAVs. Features derived from the structure of the RNA polymerase or the change in chemistry introduced by the mutation were considered; however, only a few, notably the distance from the rifampicin binding site, were found to be predictive on their own. Owing to the paucity of RAVs we used Monte Carlo cross-validation with 50 repeats to train four different machine learning models.

**Results:**

All four models behaved similarly with sensitivities and specificities in the range 0.84–0.88 and 0.94–0.97, although we preferred the ensemble of decision tree models as they are easy to inspect and understand. We showed that measuring distances from molecular dynamics simulations did not improve performance.

**Conclusions:**

It is possible to predict whether a mutation in *rpoB* confers resistance to rifampicin using a machine learning model trained on a combination of structural, chemical and evolutionary features; however, performance is moderate and training is complicated by the lack of data.

## Shareable abstract

**Machine learning works to predict whether rpoB mutations confer ripampicin resistance** https://bit.ly/3DRSi7H

## Introduction

Antimicrobial resistance is a growing global problem. This is a particular issue in tuberculosis (TB), a disease which is often fatal if untreated. In 2022, 10.6 million people developed TB and 1.3 million died [1]. TB is difficult to treat and requires multiple antibiotics to be taken for months. Drug susceptibility testing is therefore key for both effective treatment and the prevention of the spread of drug resistance. Whilst culture-based testing remains the “gold standard”, it is slow, expensive and requires considerable expertise. Nucleic acid amplification tests (NAATs), such as the Cepheid GeneXpert MTB/RIF, take a matter of hours and have been heavily subsidised, encouraging their uptake. GeneXpert MTB/RIF detects nucleotide mutations in a small region of the gene which is the target for rifampicin [2–4]. Any mutation is assumed to confer resistance to rifampicin, and since isoniazid resistance is epidemiologically associated with rifampicin resistance, resistance to isoniazid is then inferred. For many years multidrug-resistant TB has been defined as resistance to both these drugs. Since NAATs predominate, most of the ∼410 000 cases of drug-resistant TB reported in 2022 were resistant to rifampicin [1] and are likely to be resistant to isoniazid and possibly other antibiotics as well.

Whole genome sequencing (WGS) and related methods, such as targeted next generation sequencing (tNGS), have the potential to offer much greater resolution since they can detect resistance in a panel of drugs. This has been spurred on in recent years by the release of catalogues of mutations in *Mycobacterium tuberculosis*, the aetiological agent of TB, by the World Health Organization (WHO) [5–7]. Like NAATs, these approaches are inferential and so suffer from the weakness that they cannot return a result when a novel or rare mutation is detected in a gene known to be associated with resistance.

Machine-learning (ML) models can potentially address this weakness, thereby complementing WGS-based approaches. Previous ML studies have typically focused on genetic features, with some also including lineage, geographical and structural features [8–10], utilising a range of model architectures. Zhang *et al.* [11] and Kuang *et al*. [12] trained convolutional neural networks to predict resistance to various TB drugs, while Chowdhury *et al*. [13] have employed more traditional ML models incorporating structural information to predict mycobacterial capreomycin resistance. The advantage of using structural features is that they potentially contain information about the physical mechanism of drug inhibition, rather than abstracting it away as genetic features do. Carter *et al.* [14] and Karmakar *et al.* [15] both predicted pyrazinamide resistance using structurally informed models, and notably, Portelli *et al*. [16] have trained a range of ML models on structural features to predict rifampicin resistance due to mutations within *rpoB*.

**FIGURE 1:**
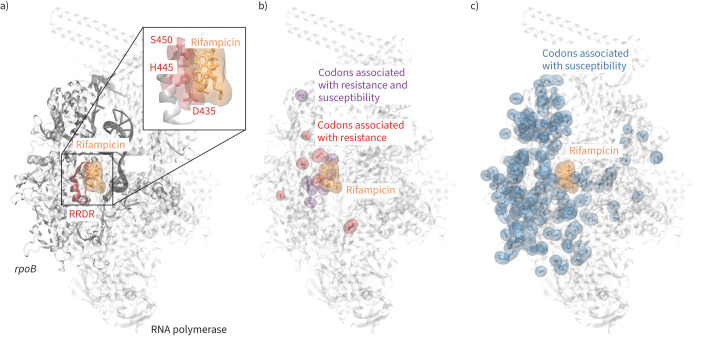
Rifampicin binds to the RNA polymerase (RNAP) and prevents the extension of nascent RNA. a) The majority of resistance-associated variants (RAVs) occur within the so-called rifampicin resistance determining region (RRDR, red) located in the RpoB subunit of the RNAP (PDB 5UH6 [17]). The three most common residues associated with resistance are Ser450, His445 and Asp435. b) The majority of RAVs are located close to the rifampicin binding site. c) Mutations not associated with resistance occur throughout the RpoB protein subunit.

Rifampicin inhibits bacterial replication by sterically occluding the extension of 2-nt to 3-nt mRNA in the RpoB subunit of the RNA polymerase (RNAP) (figure 1a) [17]. Consequently, resistance-associated variants (RAVs) within *rpoB* are predominantly located in the so-called “rifampicin resistance defining region” (RRDR), which forms the rifampicin binding interface. This contrasts with variants associated with pyrazinamide resistance, for example, which are spatially distributed more evenly in its target protein, PncA [14]. We hypothesise that this well-defined mechanism of resistance should facilitate efficient label discrimination, enabling accurate predictions with fewer features and a less complex model.

In this article, we shall determine how well a series of machine learning models can predict whether missense mutations in the *rpoB* gene result in susceptibility or instead confer resistance to rifampicin. Our models are all trained on the same Train+Test dataset, and are informed by a combination of protein structural (*e.g.* distances), chemical and evolutionary features to attempt to capture as much information as possible on how the drug binds to the protein. Since proteins are dynamic under physiological conditions, we also investigate if using distances derived from classical molecular simulations, rather than from the experimental structure, improves performance.

## Materials and methods

### Dataset

We built a dataset comprising unique missense mutations derived from 33 091 isolates collected by the CRyPTIC consortium [18]. Each isolate underwent WGS using Illumina sequencing and rifampicin susceptibility testing. The consensus genome for each sample was inferred from the short genetic read files using the Minos variant caller as incorporated into v0.12.4 of the clockwork pipeline [19, 20]. The resulting variant call files were then processed by gnomonicus (v2.5.1; https://github.com/oxfordmmm/gnomonicus), which calculated all relevant genetic variants, including translating the sequence to identify amino acid mutations. Filtering to only include samples with missense mutations in the *rpoB* gene resulted in 15 356 samples.

Binary resistant/susceptibility results were produced using a wide range of antibiotic susceptibility testing methods. The two most common were Mycobacterial Growth Indicator Tube (MGIT) and 96-well broth microdilution plate. The former is standard, whilst the latter was developed for the CRyPTIC project [20]. Owing to the challenges associated with interpreting mycobacterial growth in broth microdilution plates, we only included minimum inhibitory concentrations (MIC) where at least two independent reading methods concurred, thereby minimising measurement error [21, 22]. A research epidemiological cut-off was then applied to classify each measurement as either resistant or susceptible [20]. In total this resulted in 14 523 samples, of which 10 058 were resistant and 4465 were susceptible.

### Train+Test dataset

To generate a Train+Test dataset of mutations with labels, we constructed a catalogue using the binary phenotypes of the samples. The underlying algorithm was inspired by methodologies employed by Walker *et al*. [23] and the first and second editions of the WHO catalogue of RAVs [5, 6], where the definitive defectives algorithm [24] was used to identify and classify benign variants. Under the assumption that RAVs in *rpoB* will always induce a resistant phenotype, and to capture all possible RAVs, we used a one-tailed binomial test against an arbitrary 5% background rate. This test was conducted under the null hypothesis at 95% confidence that there is no statistically significant difference between the proportion of resistance in samples containing the mutation, when it is the only mutation present in *rpoB*, and the background rate. If the null hypothesis was accepted, the mutation was classified as susceptible (S); if rejected, it was classified as resistant (R). This approach generated a catalogue of 357 mutations. However, after selecting missense mutations that occur within the non-attenuated region of the experimental structure, the Train+Test dataset comprised 219 susceptible mutations and only 46 resistant mutations.

Best practice would be, at this point, to set aside a fraction of the dataset to use for validation; however, this is not practical here due to the paucity of mutations associated with resistance (positives). Additionally, the presence of even one or two false negatives would have a spuriously large impact on the apparent performance. Instead we opted to perform Monte Carlo cross-validation [25] using an 80:20 split whereby for each iteration 80% of the dataset is used to train the models and the performance is calculated using the remaining 20%. We arbitrarily repeat this 50 times, each time using a different (known and specified) random seed to ensure reproducibility.

### Training and hyperparameter tuning

We chose to train four different machine learning models using the Python3 scikit-learn library [26]: these were logistic regression (LR), decision tree (DT), random forest (RF) and gradient-boosted decision tree (GBDT) algorithms. Features were scaled, decision thresholds selected, and model hyperparameters tuned *via* grid searches under 5-fold stratified cross-fold validation on the Train dataset within each iteration. Given the clinical aim of minimising false negatives (very major errors (VMEs)), all models were optimised for recall. Sensitivity (*i.e.* recall) and specificity measured on the test set in each iteration were used as the primary performance metrics throughout the study: these equate to one minus the VME or major error rate, respectively.

### Determination of structural and chemical features

A range of features were generated for each mutation using the Python package sbmlcore (https://github.com/fowler-lab/sbmlcore) from an experimental structure of the *M. tuberculosis* RNAP with rifampicin bound, resolved to 3.8 Å [17]. Structural features included the distances from the C*_α_* atom of the residue in question to the centres of mass of the rifampicin and mRNA molecules, antisense and sense DNA strands, and magnesium and zinc ions, φ and ψ protein backbone angles (all calculated using MDAnalysis [27, 28]), protein secondary structure (using STRIDE) [29], the change in the number of hydrogen bond donors and acceptors, and the change in the solvent accessible surface area (SASA, using FreeSASA) [30]. Chemical features included changes on mutation in molecular weight, volume, hydropathy, isoelectric point and a chemical similarity score [31]. Additionally, we included changes in a score designed to predict whether mutations are neutral or deleterious (SNAP2) [32], as well as the results of two machine learning models that predict the change in protein stability due to a mutation (DeepDDG [33] and RasP [34]). Finally, to assess the effect of dynamics on the measured distances, we measured the minimum distances from a series of published molecular dynamics simulations of the RNAP protein [35]. To avoid outliers, the minimum distance was defined as the 5th percentile of the distances aggregated from all three simulations.

## Results

### Spatial distribution of mutations

Despite *rpoB* being an essential gene, we observe missense mutations along the entire length of the gene. Of the 46 unique resistance-associated variants, 36 are located within the RRDR (codons 426–452), with the remainder (figure 1b) close to the rifampicin binding site. In contrast, only 13 of the 219 susceptible mutations are found within the RRDR and the remainder (figure 1c) appear scattered throughout the structure of RpoB. We accordingly hypothesised that distance-based features would be predictive.

### A few features are very predictive

**FIGURE 2:**
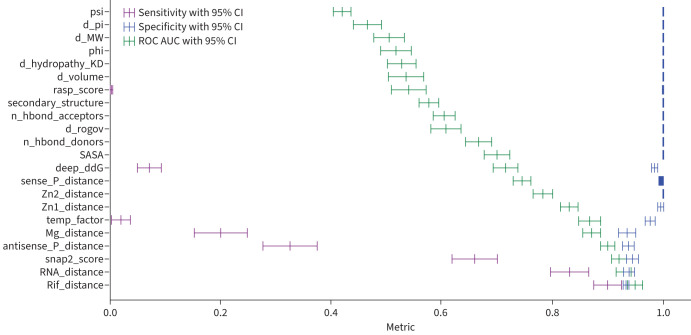
Sensitivity, specificity and area under the curve (AUC) were calculated from Monte Carlo cross-validated univariate logistic regression models, each trained on a single feature in isolation, using 50-repeat Monte Carlo cross-validation, and ordered by ROC AUC. ROC: receiver operating characteristic.

The correlation between the chosen features (supplementary figure S1) shows that many features, as expected, are confounded. This is particularly true for distances, but also applies to features representing stability (SNAP2 score) and residue flexibility (temperature factors) due to the relatively buried nature of the RRDR. We examined the predictive power of each feature by training a univariate LR model using Monte Carlo cross-validation with 50 repeats. The performance of these models (figure 2) clearly indicates that only a handful of features possess any significant predictive power in isolation – notably, any of the distances, SNAP2 score and the structural temperature factor.

**FIGURE 3:**
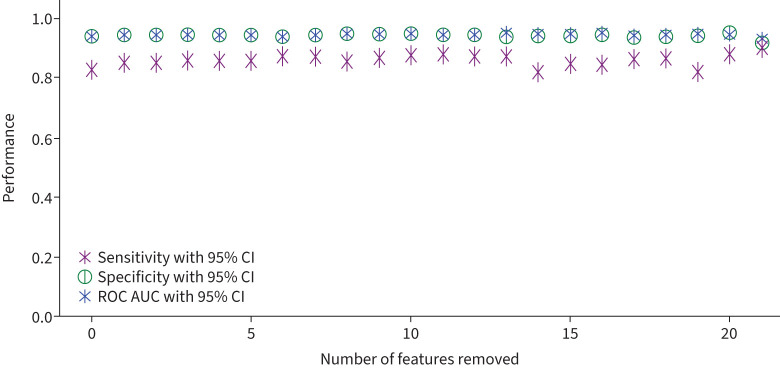
Mean sensitivity, specificity and area under the ROC curve (ROC AUC) calculated for each backwards elimination step, with sem error bars generated under 50-repeat Monte Carlo cross-validation. ROC: receiver operating characteristic.

To determine how many predictive features are required for a stable model, we performed backwards elimination of features as ordered according to the receiver operating characteristic area under the curve (ROC AUC) values of the univariate LR models (figure 2). This demonstrated one could only train on distance to rifampicin and still get robust performance (figure 3). Since several of these features are likely to interact, we chose to retain the top four most discriminatory features: these are the distances to the centres of mass of rifampicin and mRNA, the SNAP2 score and the temperature factors, whilst noting that these features are confounded to some degree.

### All models predict rifampicin resistance with moderate sensitivity

**FIGURE 4:**
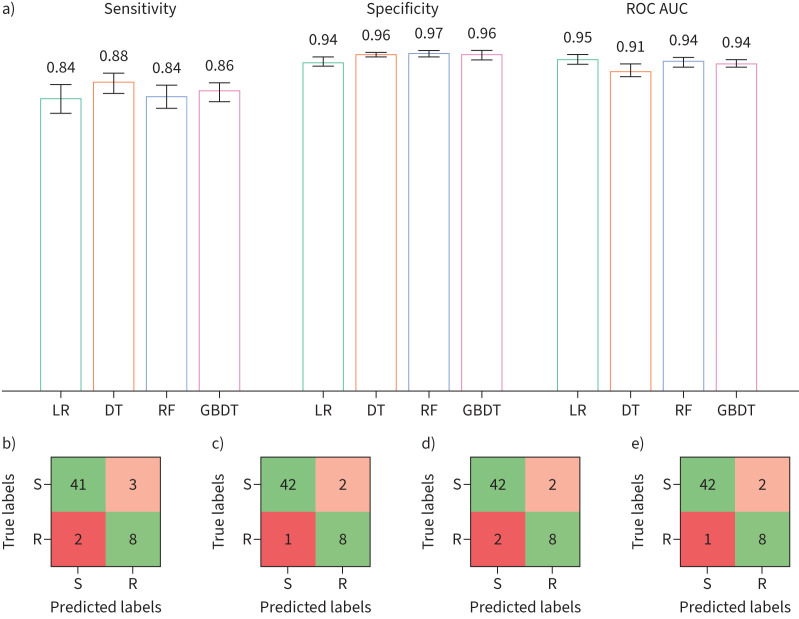
a) Mean sensitivity, specificity and area under the ROC curve (ROC AUC) values for hyperparameter tuned logistic regression (LR), decision tree (DT), random forest (RF) and gradient-boosted decision tree (GBDT) models trained under a 50-repeat Monte Carlo cross-validation protocol with 80:20 Train:Test splits. Standard error of the mean 95% confidence intervals are overlaid. All models were trained on distance to rifampicin, distance to mRNA, SNAP2 score and temperature factors. b–e) Confusion matrices showing the mean predicted true positive, false positive, true negative and false negative values for the Test sets generated in a for each model in the same order. ROC: receiver operating characteristic.

Following feature selection and hyperparameter tuning, four machine learning models (LR, DT, RF and GBDT) were trained using the above four features and validated using Monte Carlo cross-validation with 50 iterations (figure 4, table 1). All models performed similarly well in the aggregate, with mean sensitivity and specificity values in the ranges of 0.84–0.88 and 0.94–0.97, respectively. The variation in performance within each iteration was also consistent across models. Given the clear relationship between resistance and proximity to rifampicin, the simplicity and transparency of the DT (sensitivity 0.876±0.028, specificity 0.96±0.008) is particularly attractive, as it can provide effective, interpretable decisions (supplementary figure S2a). All subsequent analysis is therefore done with the ensemble of DT models.

**TABLE 1:** Mean sensitivity, specificity and area under the receiver operating characteristic curve (ROC) with standard error of the mean 95% confidence intervals for the logistic regression (LR), decision tree (DT), random forest (RF) and gradient-boosted decision tree (GBDT) models, as well as for the decision tree trained on structural data calculated from molecular dynamics trajectories (dynamic DT), all trained and tested on the same data under a 50-repeat Monte Carlo cross-validation protocol

	Sensitivity	Specificity	Area under ROC
**LR**	0.836±0.040	0.941±0.011	0.949±0.013
**DT**	0.876±0.028	0.963±0.008	0.915±0.018
**RF**	0.842±0.032	0.965±0.008	0.942±0.012
**GBDT**	0.856±0.030	0.961±0.009	0.938±0.011
**Dynamic DT**	0.835±0.033	0.956±0.009	0.895±0.018

The ensemble of DT models consistently makes six VMEs (false negatives). These predominantly involve mutations located >13 Å from the rifampicin binding site that have been catalogued as resistant: these are E207K, L731P, S493R, V262A, S582A,and L378R. However, these mutations are very rare in the clinical dataset, with each mutation present in only two samples, suggesting that these errors may be attributable to laboratory mislabeling or phenotyping errors. The model also makes a number of major errors (false positives) within 13 Å of the rifampicin binding site; however, the overall specificity is minimally affected due to the far higher number of correctly predicted susceptible mutations outside this region.

### Different models make different errors

If those six mutations were indeed a result of laboratory mislabelling, one might expect the other models to also misclassify them. However, by examining the mutations where at least one model consistently makes a mistake, we find that, despite all having similar performances, the ensembles of each of the four models all behave differently (supplementary figure S3). Closer inspection shows that the ensemble of LR models makes several major errors (L443F, S493W/L, P454S), presumably because, unlike the tree-based methods, it cannot ameliorate the proximity of these residues to rifampicin.

### Dynamical features do not improve performance

Thus far all distances have been calculated from an experimental and therefore static structure of the RNAP. *In vivo* and at physiological temperature the protein would be dynamic, and it is notable that the temperature (β) factor, which includes some element of the protein dynamics, is one of the more predictive features. We therefore hypothesised that including some measure of the dynamics might improve the discrimination of the models. We measured the minimum distance between each residue and the centre of mass of rifampicin from a set of molecular dynamics simulations [35].

**FIGURE 5:**
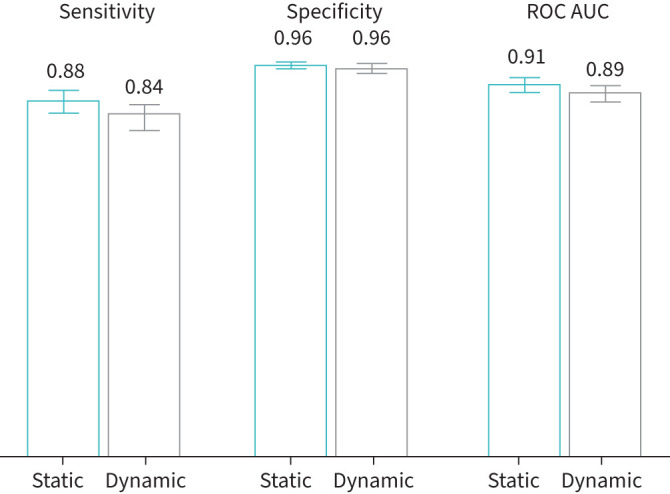
Mean sensitivity, specificity and area under the ROC curve (ROC AUC) values for hyperparameter-tuned decision trees (DT) trained under a 50-repeat Monte Carlo cross-validation protocol with 80:20 training:validation splits. Standard error of the mean 95% confidence intervals are overlaid. The models were trained on SNAP2 score, and temperature factors, as well as either distance to rifampicin (“static”) or minimum distance to rifampicin (“dynamic”), and distance to mRNA (“static”) or minimum distance to mRNA (“dynamic”). ROC: receiver operating characteristic.

**FIGURE 6:**
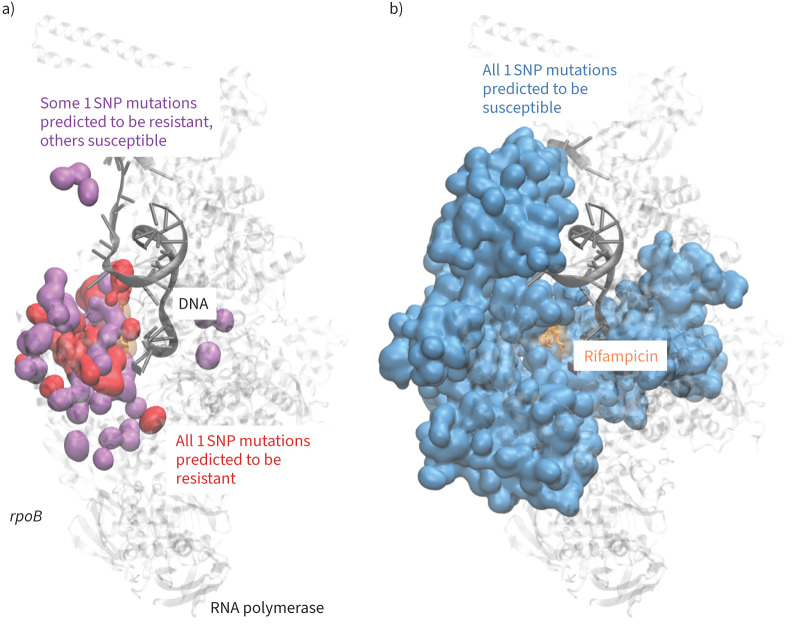
Mutations close to the rifampicin binding site are predicted to confer resistance. We used the ensemble of 50 decision tree models to predict all missense mutations in *rpoB*. a) Close to the rifampicin binding site the models predicted that all mutations at a residue (red) led to resistance, whilst the mutation of some residues could lead to resistance or have no effect (purple). b) Mutation of the majority of codons was predicted to have no effect, *i.e.* the bacterium would remain susceptible to rifampicin. SNP: single nucleotide polymorphism.

Training a DT, however, on the minimum distances to rifampicin and mRNA, the SNAP2 score, and temperature factors yielded no improvement over simply using the static distances, with a sensitivity of 0.835±0.033 and specificity of 0.956±0.009 (figure 5, table 1). With hindsight this is perhaps unsurprising, as although the magnitudes of the distances have changed, their magnitudes relative to one another remain fairly constant. Predicting all missense mutations due to SNPs (figure 6) shows how distance dominates the other features.

## Discussion

We can predict with moderate sensitivity whether individual mutations in the *rpoB* gene confer resistance to rifampicin using machine learning models trained on several structural, chemical and evolutionary features. As suggested by the spatial distribution of the mutations in the Train+Test dataset (figure 1b,c), any of the distances are moderately predictive on their own (figure 2a). SNAP2, which aims to predict the effect of mutations on a protein's function, and the temperature factor are also predictive and were therefore included as features in our models. Because there are so few resistance-conferring mutations in the Train+Test dataset, and because one must ensure that any mutation in a Test dataset is not present in the Training dataset, it was not possible to use a conventional Train/Test split. Instead, we used Monte Carlo cross-validation with 50 repeats, with each iteration using a pre-defined random seed for reproducibility [25]. All the models performed similarly; we preferred the ensemble of DT models due to their simplicity and transparency. The DT models achieved, on average, a sensitivity of 0.876±0.028 and a specificity of 0.963±0.008. Interestingly, whilst all the models behaved similarly, they tended to misclassify different mutations. To investigate if including dynamic information improves performance, we replaced the distances observed from the static crystallographic structure [17] with the minimum distances measured from several classical molecular dynamics simulations, but there was no significant effect. Including a measure of the protein dynamics will, we are sure, be important for some antibiotic targets.

To get an idea of how well our models performed compared to published models, we submitted our Train+Test dataset to the SUSPECT-RIF webserver [16]. The observed sensitivity of 0.935 was an improvement on the performance of our models which ranged from 0.84 to 0.88 and a slight decrease on their reported value of 1.00. The specificity was, however, markedly different and we observed a value of 0.293 which is far lower than seen for our models (0.94–0.97) and also less than reported on their test set. This performance suggests, unfortunately, overfitting and also illustrates that, whilst recognising that reducing the very major error rate is the primary objective, one cannot neglect the major error rate if one is to train a model that could be suitable for clinical use. We also urge researchers to make their data and code publicly available so models can be re-trained, results can be reproduced and model performance can be evaluated openly and fairly.

In practice, an ML model, such as trained here, would be used to provide some guidance when a sample is found by a WGS-pipeline to contain mutations in the RNAP that are not present in a published resistance catalogue. These mutations are therefore rare, and hence it is impractical to assess the expected performance of either model in this scenario. Instead let us consider how well the ML models perform on the mutations not in the RRDR. Since there are only 10 mutations classified as resistant not in the RRDR, in the Train+Test dataset we shall simply consider the whole dataset shorn of any mutation within the RRDR. Both our model and SUSPECT-RIF perform less well, with each model recording sensitivities and specificities of 0.432 and 0.999 and 0.700 and 0.311, respectively. The reduction in sensitivity observed for both models is likely an indication that both models, unsurprisingly, rely on a distance feature to predict resistance, and the similar specificity seen for SUSPECT-RIF is consistent with a model that has been over-trained and is over-calling resistance.

Whilst structure-based machine learning shows promise, it is most likely to succeed for drugs where the target gene is not essential as this typically leads to larger numbers of resistance- and susceptibility-associated mutations, such as pyrazinamide [14] and bedaquiline. We therefore expect similar problems to be encountered for the fluoroquinolones and isoniazid in TB where the target gene is essential. Alchemical free energy methods offer an alternative approach to machine learning; these use molecular simulation to calculate how the binding free energy of the antibiotic changes upon introduction of the mutation. These can be predictive [36, 37] but typically result in large confidence limits [35] and require many orders of magnitude more computational resources than machine learning. These methods will therefore likely have to wait a few years until the amount of computational resources required becomes feasible.

Our study has several shortcomings. By focusing on learning the binary effect (resistant/susceptible) of individual mutations we could only include samples with a single missense mutation in *rpoB* and some information will therefore have been discarded. This approach also precluded the possibility of including information from the other genes in the RNAP: in theory this could boost predictive power by allowing the models to, for example, learn about compensatory mutations [38], but that is incompatible with the approach we used here. Choosing to use structural and chemical features of individual mutations also meant the models could only learn the effects of missense mutations: nonsense mutations, insertions, deletions and promoter mutations could not be predicted. One could, of course, allow other features, such as lineage, into the models. In theory this could improve performance, but in practice it is unlikely to have much of an effect due to the dominance of the distance-based metrics. We have also assumed that the protein structure is unaltered by the missense mutations. Since RNAP is essential, it is reasonable to assume any structural changes will be small, but it is likely that some of the mutations will lead to structural changes, and these will not have been captured. As noted above, the paucity of RAVs introduces too much stochasticity into a Train/Test split and we caution that this could easily result in a “fortunate” separation of mutations leading to spuriously good performance; clearly both care and more data are needed. Finally, we have been conservative and reported performance at the level of individual mutations; this places undue weight on the rarer mutations and, if weighted according to the observed clinical prevalence, the calculated sensitivities would be higher and could be directly compared to those of the published catalogues [5, 6]. That said, this would not reflect the real-world use case, which would be making predictions on samples containing mutations that are not present in resistance catalogues. This, of course, is challenging to assess since, by definition, those mutations are unlikely to be in any Train+Test dataset. Therefore, we chose to take the simplest approach and report all metrics at the level of individual mutations. If this seems unduly conservative, we encourage the reader to apply the trained models using the data and code in the attendant GitHub repository (https://github.com/fowler-lab/predict-rifampicin-resistance).

In addition to the usual plea for more data, especially samples with rifampicin MIC as one could then train models to learn the magnitude of any shift in MIC which is likely to improve model performance, we need to explore more sophisticated machine learning model approaches. In particular, we need to explore models that, rather than learning the effect of individual mutations, can learn the structural, chemical and evolutionary features of the full genetic sequence, ideally including all the genes that make up the RNAP. This would maximise the information available to the models and would also allow nonsense mutations and insertions and deletions to be predicted (but still not promoter mutations). There is, however, no doubt that for genetics-based clinical microbiology to succeed, truly predictive methods, such as offered by machine learning, will be needed to fill the inferential gap when mutations not in any catalogue are encountered.

## Supplementary material

**Please note:** supplementary material is not edited by the Editorial Office, and is uploaded as it has been supplied by the author. Supplementary material 00952-2024.SUPPLEMENT

## References

[1] World Health Organisation. Global Tuberculosis Report 2023. Geneva, World Health Organization, 2023.

[2] Kohli M, Schiller I, Dendukuri N, et al. Xpert MTB/RIF Ultra and Xpert MTB/RIF assays for extrapulmonary tuberculosis and rifampicin resistance in adults. Cochrane Database Syst Rev 2021; 1: CD012768. doi:10.1002/14651858.CD012768.pub3 33448348 PMC8078545

[3] Mekkaoui L, Hallin M, Mouchet F, et al. Performance of Xpert MTB/RIF Ultra for diagnosis of pulmonary and extra-pulmonary tuberculosis, one year of use in a multi-centric hospital laboratory in Brussels, Belgium. PLoS ONE 2021; 16: e0249734. doi:10.1371/journal.pone.0249734 33831077 PMC8031447

[4] Weyer K, Mirzayev F, Migliori GB, et al. Rapid molecular TB diagnosis: evidence, policy making and global implementation of Xpert MTB/RIF. Eur Respir J 2013; 42: 252–271. doi:10.1183/09031936.00157212 23180585

[5] World Health Organization. Catalogue of Mutations in Mycobacterium tuberculosis complex and Their Association with Drug Resistance. 1st Edn. Geneva, World Health Organization, 2021.

[6] World Health Organization. Catalogue of Mutations in Mycobacterium tuberculosis complex and Their Association with Drug Resistance. 2nd Edn. Geneva, World Health Organization, 2023.

[7] Walker TM, Miotto P, Köser C, et al. The 2021 WHO catalogue of *Mycobacterium tuberculosis* complex mutations associated with drug resistance: a genotypic analysis. Lancet Microbe 2022; 3: e265–e273. doi:10.1016/S2666-5247(21)00301-3 35373160 PMC7612554

[8] The CRyPTIC Consortium, Yang Y, Walker TM, Walker AS, et al. DeepAMR for predicting co-occurrent resistance of *Mycobacterium tuberculosis*. Bioinformatics 2019; 35: 3240–3249. doi:10.1093/bioinformatics/btz067 30689732 PMC6748723

[9] T he CRyPTIC Consortium, Kouchaki S, Yang YY, Walker TM, et al. Application of machine learning techniques to tuberculosis drug resistance analysis. Bioinformatics 2019; 35: 2276–2282. doi:10.1093/bioinformatics/bty949 30462147 PMC6596891

[10] Sharma A, Machado E, Lima KVB, et al. Tuberculosis drug resistance profiling based on machine learning: a literature review. Braz J Infect Dis 2022; 26: 102332. doi:10.1016/j.bjid.2022.102332 35176257 PMC9387475

[11] Zhang A, Teng L, Alterovitz G. An explainable machine learning platform for pyrazinamide resistance prediction and genetic feature identification of *Mycobacterium tuberculosis*. J Am Med Inform Assoc 2021; 28: 533–540. doi:10.1093/jamia/ocaa233 33215194 PMC7936518

[12] Kuang X, Wang F, Hernandez KM, et al. Accurate and rapid prediction of tuberculosis drug resistance from genome sequence data using traditional machine learning algorithms and CNN. Sci Rep 2022; 12: 2427. doi:10.1038/s41598-022-06449-4 35165358 PMC8844416

[13] Chowdhury A, Khaledian E, Broschat S. Capreomycin resistance prediction in two species of *Mycobacterium* using a stacked ensemble method. J Appl Microbiol 2019; 127: 1656–1664. doi:10.1111/jam.14413 31419358

[14] Carter JJ, Walker TM, Walker AS, et al. Prediction of pyrazinamide resistance in *Mycobacterium*. JAC Antimicrob Resist 2024; 6: dlae037. doi:10.1093/jacamr/dlae037 38500518 PMC10946228

[15] Karmakar M, Rodrigues CH, Horan K, et al. Structure guided prediction of pyrazinamide resistance mutations in pncA. Sci Rep 2020; 10: 1875. doi:10.1038/s41598-020-58635-x 32024884 PMC7002382

[16] Portelli S, Myung Y, Furnham N, et al. Prediction of rifampicin resistance beyond the RRDR using structure-based machine learning approaches. Sci Rep 2020; 10: 18120. doi:10.1038/s41598-020-74648-y 33093532 PMC7581776

[17] Lin W, Mandal S, Degen D, et al. Structural basis of *Mycobacterium tuberculosis* transcription and transcription inhibition. Mol Cell 2017; 66: 169–179. doi:10.1016/j.molcel.2017.03.001 28392175 PMC5438085

[18] The CRyPTIC Consortium A data compendium associating the genomes of 12,289 *Mycobacterium tuberculosis* isolates with quantitative resistance phenotypes to 13 antibiotics. PLoS Biol 2022; 20: e3001721. doi:10.1371/journal.pbio.3001721 35944069 PMC9363010

[19] The CRyPTIC Consortium, Hunt M, Letcher B, Malone KM, et al. Minos: variant adjudication and joint genotyping of cohorts of bacterial genomes. Genome Biol 2022; 23: 147. doi:10.1186/s13059-022-02714-x 35791022 PMC9254434

[20] CRyPTIC Consortium. Epidemiological cut-off values for a 96-well broth microdilution plate for high-throughput research antibiotic susceptibility testing of *M. tuberculosis*. Eur Respir J 2022; 60: 2200239. doi:10.1183/13993003.00239-2022 35301246 PMC9556810

[21] Fowler PW, Gibertoni Cruz AL, Hoosdally SJ, et al. Automated detection of bacterial growth on 96-well plates for high-throughput drug susceptibility testing of *Mycobacterium tuberculosis*. Microbiology (Reading) 2018; 164: 1522–1530. doi:10.1099/mic.0.000733 30351270 PMC13037022

[22] Fowler PW, Wright C, Spiers-Bowers H, et al. A crowd of BashTheBug volunteers reproducibly and accurately measure the minimum inhibitory concentrations of 13 antitubercular drugs from photographs of 96-well broth microdilution plates. eLife 2022; 11: e75046. doi:10.7554/eLife.75046 35588296 PMC9286738

[23] Walker TM, Kohl TA, Omar SV, et al. Whole-genome sequencing for prediction of *Mycobacterium tuberculosis* drug susceptibility and resistance: a retrospective cohort study. Lancet Infect Dis 2015; 15: 1193–1202. doi:10.1016/S1473-3099(15)00062-6 26116186 PMC4579482

[24] Dorfman R. The detection of defective members of large population. Ann Math Statist 1943; 14: 436–440. doi:10.1214/aoms/1177731363

[25] Xu QS, Liang YZ. Monte Carlo cross validation. Chemom Intell Lab Sys 2001; 56: 1–11. doi:10.1016/S0169-7439(00)00122-2

[26] Pedregosa F, Varoquaux G, Gramfort A, et al. Scikit-learn: Machine Learning in Python. J Machine Learning Res 2011: 2825–2830.

[27] Michaud-Agrawal N, Denning EJ, Woolf TB, et al. MDAnalysis: a toolkit for the analysis of molecular dynamics simulations. J Comput Chem 2011; 32: 2319–2327. doi:10.1002/jcc.21787 21500218 PMC3144279

[28] Gowers R, Linke M, Barnoud J, et al. MDAnalysis: A Python Package for the Rapid Analysis of Molecular Dynamics Simulations. *In*: Proceedings of the 15th Python in Science Conference, 2016, pp. 98–105.

[29] Heinig M, Frishman D. STRIDE: a web server for secondary structure assignment from known atomic coordinates of proteins. Nucl Acid Res 2004; 32: W500–W502. doi:10.1093/nar/gkh429 PMC441567 15215436

[30] Mitternacht S. FreeSASA: an open source C library for solvent accessible surface area calculations. F1000Res 2016; 5: 189. doi:10.12688/f1000research.7931.1 26973785 PMC4776673

[31] Rogov SI, Nekrasov AN. A numerical measure of amino acid residues similarity based on the analysis of their surroundings in natural protein sequences. Protein Eng Des Sel 2001; 14: 459–463. doi:10.1093/protein/14.7.459 11522918

[32] Hecht M, Bromberg Y, Rost B. Better prediction of functional effects for sequence variants. BMC Genomics 2015; 16: Suppl. 8, S1. doi:10.1186/1471-2164-16-S8-S1 PMC4480835 26110438

[33] Cao H, Wang J, He L, et al. DeepDDG: predicting the stability change of protein point mutations using neural networks. J Chem Inf Model 2019; 59: 1508–1514. doi:10.1021/acs.jcim.8b00697 30759982

[34] Blaabjerg LM, Kassem MM, Good LL, et al. Rapid protein stability prediction using deep learning representations. eLife 2023; 12: e82593. doi:10.7554/eLife.82593 37184062 PMC10266766

[35] Brankin AE, Fowler PW. Predicting antibiotic resistance in complex protein targets using alchemical free energy methods. J Comput Chem 2022; 43: 1771–1782. doi:10.1002/jcc.26979 36054249 PMC9545121

[36] Fowler PW, Cole K, Gordon NC, et al. Robust prediction of resistance to trimethoprim in *Staphylococcus aureus*. Cell Chem Biol 2018; 25: 339–349. doi:10.1016/j.chembiol.2017.12.009 29307840

[37] Fowler PW. How quickly can we predict trimethoprim resistance using alchemical free energy methods? Interface Focus 2020; 10: 20190141. doi:10.1098/rsfs.2019.0141 33178416 PMC7653339

[38] Brunner VM, Fowler PW. Compensatory mutations are associated with increased in vitro growth in resistant clinical samples of *Mycobacterium tuberculosis*. Microb Genom 2024; 10: 001187. 38315172 10.1099/mgen.0.001187 PMC10926696

